# Is Oral Anticoagulation an Independent Predictor of Mortality in STEMI and High‐Risk NSTEMI Patients After Primary PCI? A Propensity Score‐Matched Retrospective Analysis

**DOI:** 10.1155/cdr/1295386

**Published:** 2026-09-30

**Authors:** Recep Polat, Fuat Polat, Yalçın Velibey, Osman Bolca, Göktürk İpek

**Affiliations:** ^1^ Department of Cardiology TC Saglik Bakanligi Dr Siyami Ersek Gogus Kalp ve Damar Cerrahisi Egitim ve Arastirma Hastanesi İstanbul Turkey; ^2^ Department of Cardiology Saglik Bilimleri Universitesi İstanbul Turkey

**Keywords:** antithrombotic therapy, long-term mortality, oral anticoagulation, primary percutaneous coronary intervention, ST-segment elevation myocardial infarction

## Abstract

**Background:**

Managing STEMI and high‐risk NSTEMI‐equivalent patients on chronic oral anticoagulation (OAC) undergoing primary PCI poses competing ischaemic and bleeding risks.

**Methods:**

This single‐centre retrospective study enrolled 604 consecutive STEMI/high‐risk NSTEMI patients undergoing primary PCI (2012–2023). OAC users (*n* = 100) were compared with propensity score–matched controls (*n* = 203). Primary endpoints included all‐cause mortality, bleeding, stroke, recurrent MI and rehospitalisation over a mean 3‐year follow‐up.

**Results:**

OAC users had a higher prevalence of prior ischaemic stroke/TIA (19% vs. 3%, *p* < 0.001) and known coronary artery disease (33% vs. 20%, *p* = 0.015). In‐hospital mortality was comparable (7% vs. 5.9%, *p* = 0.80). Postdischarge long‐term mortality was significantly higher in OAC users (31% vs. 14.8%, *p* = 0.001), as were access‐site haematoma (8% vs. 2%, *p* = 0.02) and minor bleeding (7% vs. 1.5%, *p* = 0.03). On multivariable Cox regression, OAC use was not an independent mortality predictor (HR 0.81, 95% CI 0.47–1.37, *p* = 0.43); age, reduced ejection fraction and chronic kidney disease were the principal determinants.

**Conclusion:**

Despite higher postdischarge mortality and bleeding, OAC use was not independently associated with mortality after adjustment. Comorbidity burden drives prognosis, emphasising comprehensive risk stratification in this population.

## 1. Introduction

Cardiovascular disease remains the leading cause of death worldwide, with acute coronary syndrome (ACS) representing its most severe manifestation [1, 2]. ST‐segment elevation myocardial infarction (STEMI) and high‐risk NSTEMI‐equivalent presentations, characterised by transmural or near‐transmural ischaemia resulting from complete or near‐complete occlusion of a coronary artery, represent the gravest clinical presentations of ACS [3, 4]. Early and effective reperfusion is the cornerstone of management in these patients, and primary percutaneous coronary intervention (PCI) is the preferred reperfusion strategy recommended by current guidelines [5, 6].

Standard antithrombotic therapy in STEMI and high‐risk NSTEMI consists of dual antiplatelet therapy (DAPT) combining aspirin with a P2Y12 receptor inhibitor [7]. However, a substantial proportion of these patients, estimated at 3%–8%, are receiving chronic oral anticoagulation (OAC) for indications such as atrial fibrillation (AF), mechanical heart valve prostheses or venous thromboembolism (VTE) at the time of presentation [8, 9]. When OAC therapy is added to standard DAPT, the resulting triple antithrombotic regimen carries a two‐ to threefold increase in major bleeding risk compared with OAC alone [10, 11]. Conversely, discontinuing OAC exposes these patients to thromboembolic events.

Despite the clinical importance of this population, randomised controlled trials addressing optimal antithrombotic strategies specifically in OAC‐treated STEMI and high‐risk NSTEMI patients are scarce. Available evidence is largely derived from registry analyses or RCTs conducted predominantly in non‐STEMI ACS settings. Differences in outcomes between patients receiving vitamin K antagonists (VKAs) and direct oral anticoagulants (DOACs) in this context remain incompletely characterised [12, 13].

The present study investigated the impact of chronic OAC use on in‐hospital and long‐term clinical outcomes in consecutive STEMI and high‐risk NSTEMI‐equivalent patients treated with primary PCI at a tertiary cardiovascular centre. Specifically, we aimed to determine whether OAC use constitutes an independent predictor of long‐term mortality and to compare bleeding and ischaemic event rates between OAC users and propensity score–matched controls.

## 2. Methods

### 2.1. Study Design and Population

This single‐centre, retrospective, observational study was conducted at a high‐volume tertiary cardiovascular centre in Istanbul, Turkey. The study was approved by the institutional Medical Specialisation and Education Committee (Approval No.: E‐28001928‐604.01.01, dated 29.09.2022) and the Ministry of Health, University of Health Sciences, and was conducted in accordance with the principles of the Declaration of Helsinki. Patient confidentiality was maintained throughout; all data were anonymised prior to analysis, and no identifying information was collected or disclosed. Informed consent was waived given the retrospective nature of the study. Patient data were obtained from the hospital′s electronic medical records system and the Turkish Ministry of Health e‐Nabız and Medula digital health platforms.

STEMI‐equivalent NSTEMI was defined by the attending interventional cardiologist before coronary angiography in patients presenting with NSTEMI who exhibited high‐risk clinical or electrocardiographic features warranting urgent coronary angiographic evaluation. The predefined criteria included (1) dynamic ST‐segment changes accompanied by haemodynamic instability or cardiogenic shock, (2) sustained ventricular arrhythmias in the setting of an ACS requiring urgent coronary angiography and revascularisation or (3) an unstable NSTEMI presentation with subsequent angiographic confirmation of total occlusion of a major coronary artery.

A total of 604 patients were identified: 100 chronic OAC users (OAC group) and 504 nonusers (control group). To balance baseline differences, propensity score matching was performed using logistic regression incorporating age, sex, diabetes, hypertension, smoking status, history of coronary artery disease, chronic kidney disease (CKD), prior stroke and anaemia. Matching was performed using a 1:2 nearest‐neighbour algorithm without replacement with a calliper of 0.05 on the propensity score scale (logit transformation). Standardised mean differences (SMDs) were calculated to assess covariate balance before and after matching and are reported in Table S1. After matching, 203 control patients were selected for comparative analysis (Figure 1).

**Figure 1:**
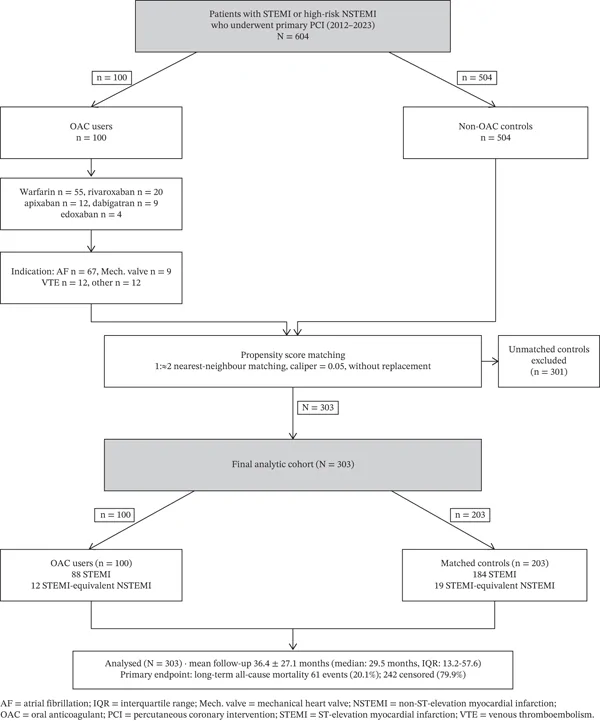
Study flow diagram depicting patient selection and propensity score matching. OAC = oral anticoagulant; STEMI = ST‐segment elevation myocardial infarction; NSTEMI = non‐ST‐segment elevation myocardial infarction; PCI = percutaneous coronary intervention.

### 2.2. Definitions

Diabetes mellitus was defined as prior use of insulin or oral antidiabetic agents or an admission blood glucose level ≥ 200 mg/dL [14]. Hypertension was defined as regular antihypertensive treatment [15]. Anaemia was defined as haemoglobin < 11 g/dL. CKD was defined as an estimated glomerular filtration rate (eGFR) < 60 mL/min [16]. Peripheral artery disease was defined as ≥ 50% stenosis on arterial Doppler or peripheral angiography. Critical coronary stenosis was defined as ≥ 50% stenosis of the left main coronary artery or ≥ 70% stenosis in non‐left‐main coronary vessels, as assessed by experienced interventional cardiologists.

Major bleeding was defined according to Thrombolysis in Myocardial Infarction (TIMI) criteria as a haemoglobin decrease ≥ 5 g/dL or requirement for red blood cell transfusion. Minor bleeding was defined as any clinically evident bleeding not meeting the criteria for major bleeding. These categories were mutually exclusive. All postdischarge bleeding events were recorded during the outpatient follow‐up period. Cerebrovascular events (CVEs) were defined as ischaemic or haemorrhagic stroke confirmed by neurologist assessment and cranial imaging. Access‐site pseudoaneurysm was diagnosed by peripheral arterial Doppler. Major adverse cardiovascular events (MACEs) comprised all‐cause death, stroke and recurrent myocardial infarction.

### 2.3. Coronary Angiography and Primary PCI Procedure

All patients underwent emergency coronary angiography using the standard Judkins technique via femoral or radial access, performed by experienced interventional cardiologists. Coronary angiographic images were evaluated using the Axiom Artis workstation (Siemens Medical Solutions, Erlangen, Germany) by two independent observers. Coronary flow was graded according to the TIMI flow classification. Primary PCI was performed according to contemporary guidelines, with balloon angioplasty and drug‐eluting or bare‐metal stent implantation as appropriate. Reperfusion success was assessed using TIMI flow grade and myocardial blush grade.

Pre‐procedural antithrombotic therapy comprised aspirin (loading dose 300 mg, maintenance 81–100 mg/day), a P2Y12 inhibitor (clopidogrel 600 mg, ticagrelor 180 mg or prasugrel 60 mg loading dose) and procedural anticoagulation (unfractionated heparin 70–100 U/kg intravenous bolus or enoxaparin 0.5 mg/kg intravenous bolus). Glycoprotein IIb/IIIa inhibitors were used selectively in patients with high thrombus burden. In OAC users, additional parenteral anticoagulation was administered regardless of the timing of the last OAC dose. Clopidogrel was preferred as the P2Y12 inhibitor in OAC users, whereas ticagrelor and prasugrel were generally avoided because of their higher bleeding risk.

### 2.4. Clinical Endpoints and Follow‐Up

The primary endpoint was long‐term all‐cause mortality after discharge. Secondary endpoints included in‐hospital mortality, major and minor bleeding events, stroke, recurrent myocardial infarction, stent thrombosis, target‐vessel revascularisation and all‐cause rehospitalisation. Follow‐up data were collected for up to 10 years through hospital records, outpatient visits and national health databases. The mean follow‐up duration was 36.4 ± 27.1 months (median, 29.5 months; interquartile range [IQR], 13.2–57.6 months; maximum, 124 months). A total of 242 patients (79.9%) were censored at their last known alive status, whereas 61 patients (20.1%) experienced the primary endpoint.

### 2.5. Statistical Analysis

Continuous variables are presented as mean ± standard deviation (SD) or median with IQR, according to their distribution. Normality was assessed using the Kolmogorov–Smirnov test. Normally distributed continuous variables were compared using the independent‐samples *t*‐test, whereas nonnormally distributed variables were compared using the Mann–Whitney *U* test. Categorical variables are presented as frequencies and percentages and were compared using the chi‐square test or Fisher′s exact test, as appropriate.

Propensity scores were estimated using logistic regression based on prespecified baseline covariates, and 1:2 nearest‐neighbour matching without replacement was performed using a calliper of 0.05 on the logit‐transformed propensity score scale. Covariate balance was assessed using SMDs.

Univariable and multivariable Cox proportional hazards regression analyses were performed to identify independent predictors of long‐term mortality. OAC use was forced into the multivariable Cox model a priori as the primary exposure of interest, regardless of its univariable *p* value. Additional covariates with *p* < 0.10 in univariable analysis were included in the multivariable model. Results are presented as hazard ratios (HRs) with 95% confidence intervals (CIs).

Procedural variables, including access site, stent implantation and glycoprotein IIb/IIIa inhibitor use, were not included in the mortality model because of the limited number of events (*n* = 61). All analyses were performed using SPSS Version 22.0 (IBM Corp., Armonk, New York, United States). All tests were two‐sided, and a *p* value < 0.05 was considered statistically significant.

## 3. Results

### 3.1. Baseline Characteristics

After propensity score matching, 100 OAC users were compared with 203 non‐OAC controls. Baseline demographic and clinical characteristics are summarised in Table 1. OAC users were slightly older (median 73 vs. 69 years, *p* = 0.15) and had significantly higher rates of prior ischaemic stroke/TIA (19% vs. 3%, *p* < 0.001) and known coronary artery disease (33% vs. 20%, *p* = 0.015). Smoking was less prevalent in OAC users (18% vs. 38%, *p* < 0.001). Other baseline characteristics, including rates of diabetes mellitus (38% vs. 39%), hypertension (69% vs. 65%), CKD (20% vs. 15%), anaemia (19% vs. 12%) and CHA_2_DS_2_‐VASc scores (2.99 vs. 2.83), did not differ significantly between groups.

**Table 1:** Baseline demographic and clinical characteristics of OAC users and propensity score–matched controls after primary PCI for STEMI.

Variable	OAC users (*n* = 100)	Non‐OAC users (*n* = 203)	*p* value
Demographic characteristics			
STEMI/NSTEMI‐equivalent breakdown, *n* (%)			
Conventional STEMI	88 (88)	184 (90.6)	0.60
STEMI‐equivalent NSTEMI	12 (12)	19 (9.4)	0.60
Age, years, median [IQR]	73 [19.75]	69 [12]	0.15
Female sex, *n* (%)	35 (35)	90 (44)	0.14
Diabetes mellitus, *n* (%)	38 (38)	79 (39)	0.90
Hypertension, *n* (%)	69 (69)	131 (65)	0.52
Current smoking, *n* (%)	18 (18)	77 (38)	< 0.001
Clinical history			
Prior CAD, *n* (%)	33 (33)	40 (20)	0.015
Chronic kidney disease, *n* (%)	20 (20)	31 (15)	0.33
Prior ischaemic stroke/TIA, *n* (%)	19 (19)	6 (3)	< 0.001
COPD, *n* (%)	12 (12)	13 (6.4)	0.12
Anaemia (Hb < 11 g/dL), *n* (%)	19 (19)	25 (12)	0.12
Peripheral artery disease, *n* (%)	6 (6)	8 (3.9)	0.40
CHA_2_DS_2_‐VASc score, mean (SD)	2.99 (1.54)	2.83 (1.47)	0.39
Anterior MI, n (%)	39 (39)	68 (33.5)	0.37
OAC agent & indication			
Warfarin, *n* (%)	55 (55)	—	
Rivaroxaban, *n* (%)	20 (20)	—	
Apixaban, *n* (%)	12 (12)	—	
Dabigatran, *n* (%)	9 (9)	—	
Edoxaban, *n* (%)	4 (4)	—	
Atrial fibrillation, *n* (%)	67 (67)	—	
Mechanical heart valve, *n* (%)	9 (9)	—	
VTE, *n* (%)	12 (12)	—	
Other, *n* (%)	12 (12)	—	
Laboratory values			
Admission INR, mean (SD)	1.83 (1.02)	1.23 (0.28)	< 0.001
Total cholesterol, mg/dL, median [IQR]	149 [51]	167 [50]	0.001
HDL cholesterol, mg/dL, median [IQR]	36 [13.5]	39 [13]	0.001
LDL cholesterol, mg/dL, mean (SD)	94.6 (33.4)	102.7 (38.4)	0.10
Triglycerides, mg/dL, mean (SD)	129.7 (65.7)	138.9 (72.1)	0.33
Serum creatinine, mg/dL, mean (SD)	1.12 (0.97)	1.03 (0.39)	0.80

*Note:* Values were expressed as median [IQR] or mean (SD) for continuous variables; *n* (%) for categorical variables. STEMI‐equivalent NSTEMI criteria: Dynamic ST changes with haemodynamic instability/cardiogenic shock, sustained ventricular arrhythmias or total coronary occlusion in unstable presentation.

Abbreviations: CAD, coronary artery disease; CKD, chronic kidney disease; COPD, chronic obstructive pulmonary disease; DAPT, dual antiplatelet therapy; HDL, high‐density lipoprotein; IQR, interquartile range; LDL, low‐density lipoprotein; MI, myocardial infarction; OAC, oral anticoagulant; SD, standard deviation; TIA, transient ischaemic attack; VTE, venous thromboembolism.

Among OAC users, 55 patients (55%) were receiving warfarin (of whom 25 had INR below the therapeutic range on admission), 20 (20%) rivaroxaban, 12 (12%) apixaban, 9 (9%) dabigatran and 4 (4%) edoxaban. The most common indication for anticoagulation was AF (67%), followed by VTE (12%), mechanical heart valve (9%) and other indications (12%). Total cholesterol and HDL cholesterol levels were significantly lower in OAC users (median 149 vs. 167 mg/dL, *p* = 0.001; median 36 vs. 39 mg/dL, *p* = 0.001), whereas other laboratory parameters did not differ significantly.

### 3.2. Procedural Characteristics

Radial artery access was used significantly more frequently in OAC users (22% vs. 0.5%, *p* < 0.001). Multivessel coronary disease was less prevalent in the OAC group (49% vs. 69.5%, *p* = 0.001). Coronary stenting was performed in 66% of OAC users versus 80.8% of controls (*p* = 0.006), whereas balloon angioplasty alone was more frequent in OAC users (15% vs. 2.5%, *p* < 0.001). CABG referral was less common in OAC users (1% vs. 6.4%, *p* = 0.04). Glycoprotein IIb/IIIa inhibitors were used less frequently in OAC users (15% vs. 28%, *p* = 0.014). Postprocedural TIMI 3 flow rates were comparable between groups (74% vs. 80%, *p* = 0.30). Procedural and in‐hospital outcome data are presented in Table 2.

**Table 2:** Procedural parameters and in‐hospital clinical outcomes.

Variable	OAC users (*n* = 100)	Non‐OAC controls (*n* = 203)	*p* value
Procedural characteristics			
Radial artery access, *n* (%)	22 (22)	1 (0.5)	< 0.001
Multivessel disease, *n* (%)	49 (49)	141 (69.5)	0.001
Stenting, *n* (%)	66 (66)	164 (80.8)	0.006
Balloon angioplasty only, *n* (%)	15 (15)	5 (2.5)	< 0.001
Medical management, *n* (%)	6 (6)	5 (2.5)	0.19
CABG referral, *n* (%)	1 (1)	13 (6.4)	0.04
Drug‐eluting stent, *n* (%)	65 (65)	163 (80)	**0.007**
GP IIb/IIIa inhibitor use, *n* (%)	15 (15)	57 (28)	0.014
Post‐PCI TIMI 3 flow, *n* (%)	74 (74)	162 (80)	0.30
Stent thrombosis, *n* (%)	4 (4)	4 (2)	0.45
In‐hospital outcomes			
Access site haematoma, *n* (%)	8 (8)	4 (2)	0.02
In‐hospital mortality, *n* (%)	7 (7)	12 (5.9)	0.80
Length of hospital stay, days, mean (SD)	5.63 (3.87)	4.78 (3.32)	0.049
ICU stay, days, mean (SD)	1.91 (1.90)	1.88 (1.81)	0.88
EF < 50*%* at discharge, *n* (%)	53 (53)	110 (54)	0.90
Discharge antithrombotic therapy			
OAC + DAPT (triple therapy), *n* (%)	**62 (67.4%)**	5 (2.6)	< 0.001
OAC + single antiplatelet, *n* (%)	**27 (29.3%)**	6 (3.1)	< 0.001
DAPT alone, *n* (%)	**3 (3.3%)**	180 (94)	< 0.001

*Note:* Discharge antithrombotic therapy denominators refer to patients surviving to discharge (*n* = 92 for OAC group; 8 OAC patients died in‐hospital and were not assigned a discharge regimen). Percentages calculated accordingly. DES *p* value corrected from 1.00 to 0.007 (chi‐square test; in bold). Procedural variables shown in this table (access site, stenting approach and GP IIb/IIIa inhibitor use) were not included in the multivariable Cox mortality model owing to the limited number of events (*n* = 61) and risk of overfitting. Their potential contribution to outcome differences is discussed in the Discussion section.

Abbreviations: CABG, coronary artery bypass grafting; DAPT, dual antiplatelet therapy; DES, drug‐eluting stent; EF, ejection fraction; OAC, oral anticoagulant.

### 3.3. In‐Hospital and Discharge Outcomes

Access site haematoma was significantly more frequent in OAC users (8% vs. 2%, *p* = 0.02). Mean length of hospital stay was longer in OAC users (5.63 vs. 4.78 days, *p* = 0.049). In‐hospital mortality did not differ significantly between groups (7% vs. 5.9%, *p* = 0.80), and no in‐hospital mortality attributable to bleeding was recorded in either group. The majority of OAC users were discharged on triple antithrombotic therapy (OAC + DAPT: 67%; OAC + single antiplatelet: 29%), whereas nearly all controls received DAPT (94%).

### 3.4. Long‐Term Outcomes

Over a mean follow‐up of approximately 3 years, long‐term all‐cause mortality after discharge was significantly higher in OAC users than in controls (31% vs. 14.8%, *p* = 0.001). The Kaplan–Meier survival analysis demonstrated a sustained and progressive divergence in survival curves between OAC users and non‐OAC controls throughout the follow‐up period, with OAC users showing markedly lower overall survival probability (approximately 71% vs. 85% at 5 years; log‐rank *p* = 0.001) (Figure 2). The median time to postdischarge death was longer in OAC users (913 vs. 395 days, *p* = 0.008). Postdischarge stroke was more frequent in OAC users (4% vs. 0.5%, *p* = 0.008), and postdischarge minor bleeding was also higher (7% vs. 1.5%, *p* = 0.03). Rates of recurrent MI (4% vs. 4.9%, *p* = 0.69), target vessel revascularisation (5% vs. 6.4%, *p* = 0.80) and overall rehospitalisation (25% vs. 26.6%, *p* = 0.89) were comparable between groups. Long‐term outcomes and Cox regression results are summarised in Table 3.

**Figure 2:**
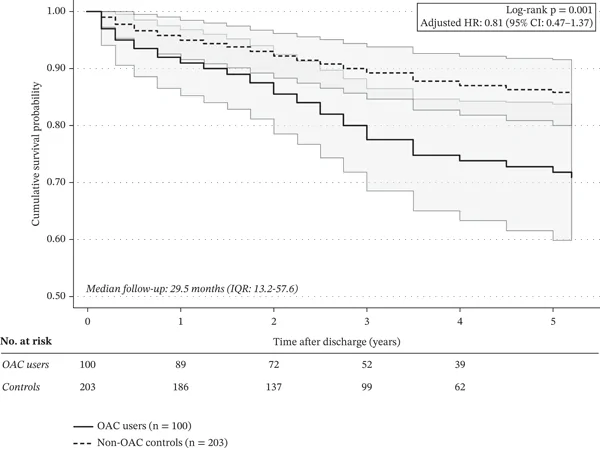
Kaplan–Meier survival curves for long‐term all‐cause mortality after discharge, comparing OAC users (*n* = 100) and propensity score–matched non‐OAC controls (*n* = 203). Log‐rank *p* = 0.001.

**Table 3:** Long‐term clinical outcomes and Cox proportional hazards regression analysis for all‐cause mortality.

Variable	OAC users (*n* = 100)	Non‐OAC controls (*n* = 203)	*p* value
Long‐term outcomes (median 29.5 months**)**			
Long‐term all‐cause mortality, *n* (%)	31 (31)	30 (14.8)	0.001
Postdischarge stroke/CVE, *n* (%)	4 (4)	1 (0.5)	0.008
Postdischarge bleeding (any), *n* (%)	7 (7)	3 (1.5)	0.03
Minor bleeding (nonoverlapping subcategory)^a^, *n* (%)	4 (4)	1 (0.5)	0.06
Major bleeding (nonoverlapping subcategory)^a^, *n* (%)	3 (3)	2 (1.0)	0.24
Recurrent MI, *n* (%)	4 (4)	10 (4.9)	0.69
Target vessel revascularisation, *n* (%)	5 (5)	13 (6.4)	0.80
All‐cause rehospitalisation, *n* (%)	25 (25)	54 (26.6)	0.89
Median time to postdischarge death, days [IQR]	913 [1613]	395 [615]	0.008

^a^Minor and major bleeding categories are mutually exclusive subcategories of postdischarge bleeding (any). Major bleeding: TIMI criteria (Hb decrease ≥ 5 g/dL or RBC transfusion required). Minor bleeding: Any clinically evident bleeding.

**Table 4:** Multivariable Cox regression—independent predictors of long‐term mortality.

Variable	Unadjusted HR	95% CI	Adjusted HR	*p* value
Age (per 1‐year increase)	1.06	1.03–1.09	1.06 (1.02–1.10)	0.001
Decreased EF (per 10% increase)	0.96	0.94–0.98	0.95 (0.93–0.98)	< 0.001
Chronic kidney disease	2.46	1.41–4.29	2.16 (1.11–4.23)	0.023
Diabetes mellitus	2.07	1.24–3.45	0.89 (0.46–1.71)	0.72
OAC use	0.81	0.47–1.37	0.81 (0.47–1.37)^a^	0.43

*Note:* OAC use was forced into the multivariable Cox model a priori as the primary exposure of interest, regardless of univariable *p* value, per standard epidemiological practice for observational studies. Adjusted HR 0.81 (95% CI 0.47–1.37, *p* = 0.43) confirms that OAC use remains a nonsignificant predictor after full adjustment. The adjusted HR of 0.81 (95% CI 0.47–1.37) is consistent with both clinically meaningful benefit and harm from OAC use; the study was underpowered to exclude either. This finding should not be interpreted as demonstrating that OAC is neutral with respect to mortality.

Abbreviations: CI, confidence interval; EF, ejection fraction; HR, hazard ratio; OAC, oral anticoagulant.

### 3.5. Predictors of Long‐Term Mortality

In univariable Cox regression analysis, age (HR 1.06 per year, 95% CI 1.03–1.09, *p* < 0.001), diabetes mellitus (HR 2.07, 95% CI 1.24–3.45, *p* = 0.005), CKD (HR 2.46, 95% CI 1.41–4.29, *p* = 0.002), total cholesterol (HR 0.99, *p* = 0.048) and ejection fraction (HR 0.96 per 10% increase, 95% CI 0.94–0.98, *p* < 0.001) were significantly associated with long‐term mortality. OAC use did not reach significance in univariable analysis (HR 0.81, 95% CI 0.47–1.37, *p* = 0.43) Table 4. In the multivariable model with OAC use forced into the model a priori, OAC use remained a nonsignificant predictor of long‐term mortality (adjusted HR 0.81, 95% CI 0.47–1.37, *p* = 0.43). A sensitivity analysis restricted to conventional STEMI patients only (*n* = 272) yielded consistent results (adjusted HR for OAC use: 0.79, 95% CI 0.45–1.40, *p* = 0.42; Table S2), supporting the robustness of the primary findings.

In multivariable Cox regression, three variables emerged as independent predictors of long‐term all‐cause mortality: older age (HR 1.06 per year, 95% CI 1.023–1.10, *p* = 0.001), reduced left ventricular ejection fraction (HR 0.95 per 10% increase, 95% CI 0.93–0.98, *p* < 0.001) and the presence of CKD (HR 2.16, 95% CI 1.11–4.23, *p* = 0.023). As illustrated in the forest plot (Figure 3), OAC use remained a nonsignificant predictor after multivariable adjustment (HR 0.81, 95% CI 0.47–1.37, *p* = 0.43), as did diabetes mellitus (*p* = 0.72), smoking (*p* = 0.65), female sex (*p* = 0.29†), hypertension (*p* = 0.93†), prior ischaemic stroke (*p* = 0.91†), history of CAD (*p* = 0.29†), anterior MI (*p* = 0.28†), multivessel disease (*p* = 0.45†) and stent length (p not entered†). Variables marked † did not reach *p* < 0.10 in univariable analysis and were, therefore, not entered into the multivariable model.

**Figure 3:**
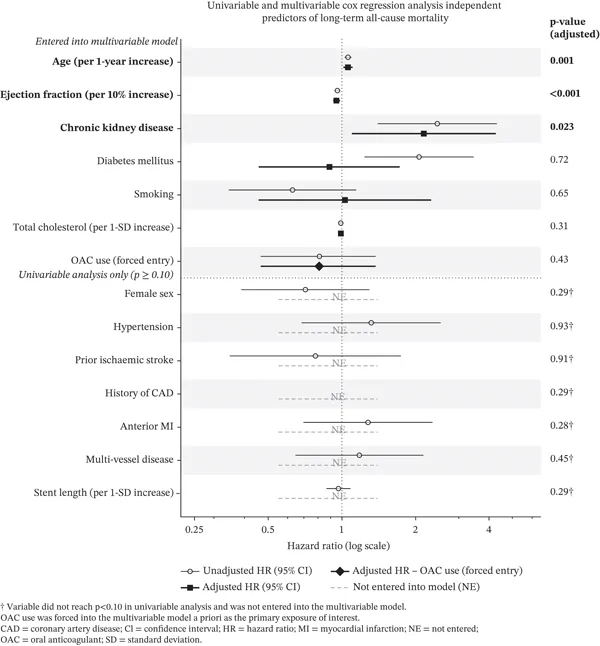
Forest plot of multivariable Cox proportional hazards regression analysis showing independent predictors of long‐term mortality. HR = hazard ratio; CI = confidence interval; CKD = chronic kidney disease; EF = ejection fraction.

## 4. Discussion

In this propensity score–matched, single‐centre retrospective study of 303 patients (100 OAC users and 203 controls) with STEMI or STEMI‐equivalent high‐risk NSTEMI treated with primary PCI, we observed that OAC users had significantly higher long‐term all‐cause mortality after discharge and higher rates of minor bleeding and stroke. However, OAC use itself was not an independent predictor of long‐term mortality in multivariable analysis; age, reduced ejection fraction and CKD were the dominant prognostic determinants. However, the absence of a detected independent association between OAC use and mortality should not be interpreted as evidence of a neutral effect. The adjusted HR of 0.81 (95% CI 0.47–1.37) spans a range consistent with both a clinically meaningful survival benefit and clinically meaningful harm from OAC use, and the study was underpowered to exclude either possibility. These findings must, therefore, be interpreted cautiously and within the constraints of the study design.

An important conceptual consideration in interpreting our findings is confounding by indication. OAC use in this population is not an isolated pharmacological exposure but rather a marker of underlying cardiovascular and thromboembolic risk conditions. AF, which accounted for 67% of OAC indications in our cohort, is itself an independent risk factor for stroke, heart failure and death. Mechanical heart valve disease and VTE similarly represent conditions associated with adverse cardiovascular prognosis. Consequently, the observed mortality excess in OAC users is likely substantially attributable to the underlying indications for anticoagulation rather than to OAC per se. Our study cannot fully disentangle the independent effect of OAC from the prognostic impact of the conditions for which it is prescribed. Although AF was included as an indirect matching variable through its clinical correlates (prior stroke, CAD), formal adjustment for OAC indication was not possible given the limited sample size. This should be considered a major limitation of the present work.

The reported prevalence of chronic OAC use among STEMI patients varies between 3% and 8% across registries [8, 17]. The present cohort aligns with this range, with AF being the predominant OAC indication (67%), consistent with the epidemiological trajectory of an ageing population with increasing AF burden. The more complex comorbidity profile of OAC users, older age, greater prevalence of prior CVE and known CAD mirrors findings from previous registries and likely accounts for the higher crude mortality observed in this group.

In‐hospital mortality did not differ significantly between OAC users and controls (7% vs. 5.9%, *p* = 0.80) in our study. This finding contrasts with some earlier observational data, such as the RICO survey by Oudot et al., which reported higher in‐hospital adverse event rates in OAC users, partly attributed to lower reperfusion rates in that population [17]. In our cohort, all patients underwent primary PCI, which likely attenuated differences in short‐term outcomes. This observation supports the principle that expeditious reperfusion therapy should not be withheld from OAC‐treated patients presenting with STEMI.

The significantly higher long‐term mortality in the OAC group (31% vs. 14.8%, *p* = 0.001) is consistent with the higher burden of comorbidities rather than a direct harmful pharmacological effect of OAC. Indeed, OAC use did not reach statistical significance even in univariable Cox analysis, suggesting that age, reduced EF and CKD, not OAC per se, are the principal drivers of mortality. This contrasts with findings from Stepinska et al., who identified OAC use as an independent predictor of long‐term mortality in a European multicentre registry [18]. The discrepancy may be explained by differences in patient selection, the proportion receiving adequate reperfusion therapy and differences in follow‐up management.

Bleeding complications represent a central concern in this patient population. In our study, access site haematoma was more frequent in OAC users (8% vs. 2%, *p* = 0.02), reflecting the higher procedural anticoagulant burden. However, these events were predominantly minor and localised to the access site, without haemodynamic consequences or mortality impact. The greater use of radial access in OAC users (22% vs. 0.5%) in our series reflects contemporary clinical practice aimed at mitigating vascular complications [19]. Postdischarge bleeding was higher in OAC users (7% vs. 1.5%), a finding consistent with data from Stepinska et al. [18] and from large meta‐analyses reporting increased bleeding risk with triple antithrombotic therapy, particularly in warfarin‐treated patients [20].

Procedural differences between groups, including more frequent radial access (22% vs. 0.5%), lower stenting rates (66% vs. 80.8%), more frequent balloon angioplasty alone (15% vs. 2.5%) and less use of GP IIb/IIIa inhibitors (15% vs. 28%) in OAC users, likely reflect deliberate clinical decisions to minimise bleeding risk in patients anticipating triple antithrombotic therapy, rather than constituting independent prognostic determinants. Notably, OAC users had a lower prevalence of multivessel disease (49% vs. 69.5%) yet experienced worse long‐term survival, further supporting the primacy of comorbidity burden over procedural complexity in driving outcomes. Nonetheless, these procedural variables were not formally included in the multivariable mortality model, and it cannot be excluded that they contribute to outcome differences.

The majority of OAC users in our cohort were discharged on triple antithrombotic therapy (OAC + DAPT), which is associated with a two‐ to threefold increase in bleeding compared with OAC alone [10, 11]. Current ESC guidelines advocate minimising the duration of triple therapy and transitioning to dual therapy (OAC + single antiplatelet agent) after the periprocedural period [21]. In our cohort, warfarin recipients who were discharged on triple therapy constituted the majority of patients experiencing postdischarge bleeding, which may partly reflect subtherapeutic INR control, consistent with established data on the relationship between INR variability and bleeding risk [22]. DOACs, which constituted 45% of the OAC group, are associated with lower rates of bleeding than warfarin when combined with antiplatelet agents [23, 24].

Interestingly, OAC users had significantly lower total cholesterol and HDL cholesterol levels. This finding may reflect the metabolic effects of warfarin on lipid metabolism, as described by Aloi et al. [25], or may indicate lower statin efficacy in this group. Postdischarge stroke rates were numerically higher in OAC users (4% vs. 0.5%, *p* = 0.008), although this likely reflects the higher baseline embolic risk from AF rather than a treatment failure, and is consistent with the expectation that OAC‐treated patients have pre‐existing thrombotic risk factors.

Several limitations of this study merit consideration. First, the retrospective, single‐centre design limits the generalisability of the findings and introduces the possibility of selection and information bias. Second, although propensity score matching was performed, several clinically relevant baseline variables remained imbalanced after matching, including prior ischaemic stroke/TIA (SMD = 0.54), prior coronary artery disease (SMD = 0.31), current smoking (SMD = 0.44), age (SMD = 0.22) and anaemia (SMD = 0.19), as shown in Table S1. These residual imbalances indicate that propensity score matching did not fully eliminate baseline differences and may have resulted in residual confounding. Third, confounding by indication remains an important concern, as chronic OAC use is largely driven by underlying conditions such as AF, whose independent effects on long‐term prognosis cannot be completely separated from the effect of OAC therapy in this observational analysis.

The OAC group was smaller than the control group, reducing statistical power for subgroup analyses, and the unequal distribution of individual DOAC subtypes precluded reliable comparisons among different OAC regimens. Furthermore, antithrombotic treatment changes during follow‐up, including de‐escalation from triple to dual therapy and switching between DOAC agents, were not systematically captured, resulting in potential time‐varying exposure misclassification. Heparin doses administered during primary PCI were not recorded in the electronic database. In addition, out‐of‐hospital event ascertainment relied partly on national health information systems, which may not have been fully accessible for all patients.

Finally, the absence of an independently significant association between OAC use and mortality should not be interpreted as evidence of no effect. Given the limited number of deaths and the heterogeneity of OAC indications, the study may have had insufficient statistical power to detect a modest association. Therefore, the findings should be interpreted cautiously, particularly in light of the residual baseline imbalances and potential for unmeasured and indication‐related confounding.

## 5. Conclusion

Among patients with STEMI or STEMI‐equivalent high‐risk NSTEMI undergoing primary PCI, chronic OAC therapy was associated with higher long‐term all‐cause mortality and postdischarge bleeding rates compared with propensity score–matched controls. However, after multivariable adjustment, OAC use was not independently associated with long‐term mortality. The findings do not establish a causal relationship between chronic OAC therapy and mortality, and clinically meaningful benefit or harm cannot be excluded. Age, reduced left ventricular ejection fraction and CKD were the principal independent determinants of long‐term prognosis. These findings highlight the importance of comprehensive assessment of comorbidities and individualised antithrombotic management in OAC‐treated patients presenting with STEMI or high‐risk NSTEMI. Prospective studies are warranted to further clarify the prognostic impact of chronic OAC therapy and to inform antithrombotic management in this high‐risk population.

## Author Contributions

R.P. conceived and designed the study and performed data collection and extraction from electronic health records. F.P. conducted the statistical analyses and drafted the initial manuscript. F.P., G.İ. and Y.V. critically revised the manuscript for important intellectual content. G.İ. and O.B. supervised the study and provided senior oversight. All authors contributed to data interpretation.

## Funding

No funding was received for this manuscript.

## Disclosure

All authors reviewed and approved the final manuscript and agree to be accountable for all aspects of the work.

## Ethics Statement

The study was conducted in accordance with the principles of the Declaration of Helsinki and approved by the Institutional Committee of TC Saglik Bakanligi Dr Siyami Ersek Gogus Kalp ve Damar Cerrahisi Egitim ve Arastirma Hastanesi, in collaboration with Sağlık Bilimleri Üniversitesi (Approval No: E‐28001928‐604.01.01; date: September 29, 2022). All procedures adhered to ethical standards to ensure participant safety, privacy and data security.

## Consent

Due to the retrospective nature of this study, the requirement for written informed consent for publication was waived by the Institutional Committee of TC Saglik Bakanligi Dr Siyami Ersek Gogus Kalp ve Damar Cerrahisi Egitim ve Arastirma Hastanesi, in collaboration with Sağlık Bilimleri Üniversitesi (Approval No: E‐28001928‐604.01.01; date: 29 September 2022). All data were anonymised to protect patient confidentiality, and no personally identifiable information is disclosed in this publication.

## Conflicts of Interest

The authors declare no conflicts of interest.

## Supporting Information

Additional supporting information can be found online in the Supporting Information section.

## Supporting information


**Supporting Information 1** Table 1: Standardised mean differences (SMDs) for all baseline covariates before and after propensity score matching, enabling assessment of covariate balance across the OAC and control groups.

**Supporting Information 2** Table 2: The results of a sensitivity analysis restricted to patients with conventional STEMI only (*n* = 272), excluding the 31 STEMI‐equivalent NSTEMI cases, and the primary Cox regression findings remain consistent in this subgroup.

## Data Availability

The research presented in this manuscript does not involve the development or utilisation of any custom software applications or specific code. As such, there is no code availability associated with this study. The analysis and findings rely on standard statistical methods and commercially available software tools (SPSS Version 25.0). For any inquiries related to the methodology or data analysis, please contact the corresponding author. All relevant data supporting the findings of this study are available upon request and will be provided by the corresponding author.
