# Supplementary material for: Is Oral Anticoagulation an Independent Predictor of Mortality in STEMI and High‐Risk NSTEMI Patients After Primary PCI? A Propensity Score‐Matched Retrospective Analysis
Source: Cardiovasc Ther. 2026 Sep 30;2026:1295386. doi: 10.1155/cdr/1295386 (PMC13627984; doi:10.1155/cdr/1295386)
Supplement: Supplementary file 1 [file CDR-2026-1295386-s001.docx]

**Supplementary Table S2.** Sensitivity Analysis: Conventional STEMI Patients Only (n=272)

| **Variable** | **Unadjusted HR (95% CI)** | **Adjusted HR (95% CI)** | **p-value** |
| --- | --- | --- | --- |
| *Sensitivity analysis* | | | |
| OAC use | 0.78 (0.44–1.37) | 0.79 (0.45–1.40) | 0.42 |
| Age (per 1-year increase) | 1.06 (1.02–1.10) | 1.06 (1.02–1.10) | 0.003 |
| Decreased EF (per 10% increase) | 0.96 (0.93–0.98) | 0.95 (0.93–0.98) | <0.001 |
| Chronic kidney disease | 2.51 (1.38–4.56) | 2.21 (1.09–4.48) | 0.028 |

*Sensitivity analysis restricted to patients with conventional STEMI (n=272: 88 OAC users, 184 matched controls), excluding 31 STEMI-equivalent NSTEMI patients. Results are consistent with the primary analysis. The adjusted HR for OAC use (0.79, 95% CI 0.45–1.40) is consistent with both clinically meaningful benefit and harm; the absence of a statistically significant association does not exclude a real effect given the limited sample size.*
