# Supplementary material for: Is Oral Anticoagulation an Independent Predictor of Mortality in STEMI and High‐Risk NSTEMI Patients After Primary PCI? A Propensity Score‐Matched Retrospective Analysis
Source: Cardiovasc Ther. 2026 Sep 30;2026:1295386. doi: 10.1155/cdr/1295386 (PMC13627984; doi:10.1155/cdr/1295386)
Supplement: Supplementary file 2 [file CDR-2026-1295386-s002.docx]

**Supplementary Table S1.** Standardised Mean Differences (SMD) Before and After Propensity Score Matching

| **Variable** | **Before PSM OAC (n=100)** | **Before PSM Control (n=504)** | **SMD Before** | **After PSM Control (n=203)** | **SMD After** |
| --- | --- | --- | --- | --- | --- |
| Age (years) | 73 [19.75] | 64 [16] | 0.41 | 69 [12] | 0.22 |
| Female sex | 35% | 36% | 0.02 | 44% | 0.18 |
| Diabetes mellitus | 38% | 37% | 0.02 | 39% | 0.02 |
| Hypertension | 69% | 60% | 0.19 | 65% | 0.08 |
| Current smoking | 18% | 41% | 0.57 | 38% | 0.44* |
| Prior CAD | 33% | 15% | 0.44 | 20% | 0.31* |
| Chronic kidney disease | 20% | 12% | 0.22 | 15% | 0.13 |
| Prior stroke / TIA | 19% | 2% | 0.56 | 3% | 0.54* |
| Anaemia (Hb <11) | 19% | 11% | 0.23 | 12% | 0.19 |

*SMD >0.20 after matching indicates residual imbalance. Variables marked * (prior stroke/TIA SMD=0.54, smoking SMD=0.44, prior CAD SMD=0.31) represent clinically important residual imbalances for which propensity score matching did not achieve adequate control. These imbalances reflect the inherent clinical heterogeneity of patients requiring OAC and represent potential sources of residual confounding; the adjusted results should be interpreted accordingly. Additional residual imbalances were observed for age (SMD=0.22) and anaemia (SMD=0.19). PSM = propensity score matching; SMD = standardised mean difference.*
